# Cost-effectiveness analysis of enzyme replacement therapy for the treatment of Chinese patients with fabry disease: a Markov model

**DOI:** 10.3389/fphar.2025.1546018

**Published:** 2025-03-11

**Authors:** Yueyang Huang, Hongmei Yuan, Zhe Huang

**Affiliations:** School of Business Administration, Shenyang Pharmaceutical University, Shenyang, China

**Keywords:** fabry disease, enzyme replacement therapy, agalactosidase-α, Markov model, incremental cost-effectiveness ratio, sensitivity analysis

## Abstract

**Background:**

Fabry disease (FD) is an X-chromosome-linked genetic disorder. Currently, the main treatments for FD include disease-nonspecific and disease-specific treatments. Nonspecific treatment involves symptomatic management of organ involvement. On the other hand, disease-specific treatment is to regulate the activity of the corresponding enzymes, which is targeted. Among them, enzyme replacement therapy (ERT) is a classical therapy. Several studies have demonstrated the significant ameliorative effect of agalactosidase-α (ALTA-a) on cardiac and renal function in patients with FD. Despite the excellent clinical performance, there are limited pharmacoeconomic studies on ERT for FD worldwide.

**Objective:**

The aim of this study was to analyze the cost-effectiveness of ERT for FD in China from the perspective of the healthcare system.

**Methods:**

We constructed a five-state Markov model based on the disease characteristics of FD. The modeling period was 1 month. The time horizon was 3 years. The willingness-to-pay threshold was chosen as 1-3 times the gross national product (GDP) *per capita*. The incremental cost-effectiveness ratio (ICER) was calculated from the base case analysis, and one-way sensitivity analysis and probabilistic sensitivity analysis were performed.

**Results:**

The ICER value is ¥148071.95/QALY, which is between 1-3 times GDP *per capita*. The sensitivity analysis showed that the cost of ALTA-a had a significant effect on ICER and proved the stability of the results.

**Conclusion:**

ERT therapy is a cost-effective program compared to “No ERT” therapy.

## 1 Introduction

Fabry disease (FD), also known as Anderson-Fabry disease, is an X-chromosome-linked genetic disorder. It is a multisystem, progressive lysosomal storage disorder caused primarily by mutations in the galactosidase alpha (GLA) gene (Spada et al., 2006). As the second most prevalent lysosomal storage disease to date after Gaucher disease (Mallett et al., 2020), the population prevalence of FD ranges from 1/117,000 to 1/40,000 (Vardarli et al., 2020), and in newborns this value ranges from 1/8882 to 1/1250 (Linhart et al., 2020). The pathogenesis of FD is a partial or total loss of α-galactosidase A (α-Gal A) activity due to mutations in the GLA gene, resulting in the accumulation of large amounts of its metabolic substrate in various organs and tissues of the body, causing a series of organ pathologies to occur (Vardarli et al., 2020). Without timely and effective treatment, patients often die due to serious complications such as renal failure, heart failure, cardiac arrhythmia and stroke (Turkmen and Baloglu, 2020). As a rare disease typically inherited from the X chromosome, male patients with FD are more likely to develop symptoms of the disease and the above complications more readily and earlier compared to females (Turkmen and Baloglu, 2020). Life expectancy has been reported to be reduced by 15–20 years for male patients with FD and 6–10 years for female patients (Hagège et al., 2019). The therapeutic goals of FD are to slow disease progression, improve quality of life, reduce the incidence of associated complications, and prolong patient survival. Currently, the main treatments for FD include disease non-specific and disease-specific treatments. Non-specific treatment refers to the treatment of specific symptoms, such as renal lesions, cardiac lesions and neurological symptoms, etc. This type of treatment can effectively relieve the symptoms, but does not address the underlying problem of enzyme deficiency and substrate accumulation. These treatments are effective in relieving symptoms, but do not address the underlying problems of enzyme deficiency and substrate accumulation. Specific therapies achieve relief of FD symptoms mainly by targeting and regulating the activity of the corresponding enzymes (Bernardes et al., 2020; Lenders and Brand, 2021; Simonetta et al., 2020). Among them, Enzyme replacement therapy (ERT) is the first-line treatment option for FD recognized by guidelines and consensus in most regions of the world (Germain et al., 2019; Hopkin et al., 2016; Biegstraaten et al., 2015; Ortiz et al., 2018), which effectively relieves patients' pain and gastrointestinal symptoms, improves cardiac hypertrophy, and stabilizes renal function by supplementing the lack of α-Gal A in the patients' body, and reduces substrate accumulation, thus improving the patients' quality of life (Germain et al., 2019; Anderson et al., 2014). And ERT drugs mainly include agalactosidase-α(ALTA-a) (Garman and Garboczi, 2004) and agalactosidase-β(ALTA-b) (McCafferty and Scott, 2019), which have demonstrated excellent efficacy and safety in many FD-related studies (Ramaswami et al., 2006; Beck et al., 2015; Beck et al., 2004; Tahir et al., 2007; Germain et al., 2007). Among them, ALTA-a entered the national health insurance drug catalogue through health insurance negotiations in 2021. Comparison of the economics of treatment regimens is important given the significant reduction in ERT drug costs and increased drug accessibility. However, there are limited pharmacoeconomic evaluation studies (Rombach et al., 2013) of FD treatments globally, and direct comparisons of the economics of specific and non-specific therapies are urgently needed. Therefore, this study conducted a cost-effectiveness analysis of ALTA-a treatment for Chinese FD patients from the perspective of the healthcare system to inform the decision-making of relevant healthcare organizations.

## 2 Methods

### 2.1 Target population

In this study, we chose male FD patients and female patients with more severe symptoms because of the different severity of the disease in male and female patients, the greater susceptibility of male patients to the disease, and the greater impact on life expectancy (Turkmen and Baloglu, 2020; Hagège et al., 2019).

### 2.2 Perspective

According to the purpose of this paper, we choose the healthcare system as the research perspective.

### 2.3 Interventions and comparators

In this study, the control protocol was the corresponding symptomatic treatment. We chose three typical symptoms of FD as treatment targets, including: pain, cardiac impairment and renal lesions. The therapeutic drugs chosen for these symptoms were: (1) pain: carbamazepine; (2) cardiac impairment: angiotensin-converting enzyme inhibitor analogue - captopril; and (3) renal lesions: angiotensin receptor antagonist analogue - chlorosartan. Carbamazepine was administered orally twice daily at 100 mg. Captopril was administered orally three times daily at 12.5 mg. Chlorosartan was administered orally once daily at 100 mg. ERT treatment was chosen as the intervention option, specifically the use of ALTA-a. It was administered intravenously at a dosage of 0.2 mg/kg every 2 weeks. According to data published by the Chinese National Nutrition Survey, the average weight of Chinese adult males is 66.2 kg, and the average weight of adult women in China is 58.3 kg.

### 2.4 Model structure

**FIGURE 1:**
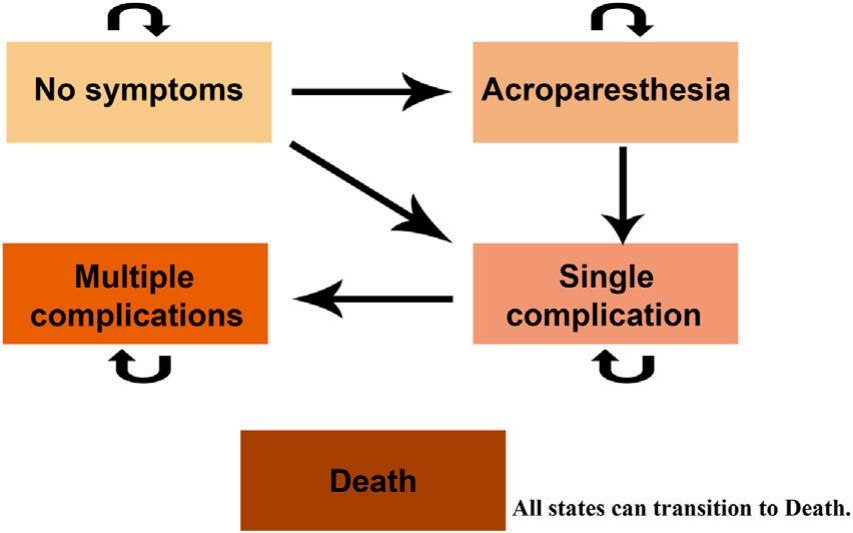
Markov model diagram.

In this study, a Markov model was developed using previous research as a reference (Rombach et al., 2013). Five health states were established according to the characteristics of the symptoms of the disease, namely, “No symptoms”, “Acroparesthesia”, “Single complication”, “Multiple complications” and “Death”. The “Single complication” state means that the patient has one of the three typical symptoms (mentioned in 2.3). The “Multiple complications” state is when the patient has two or three of the three typical symptoms. Patients in any other state are able to move to the “Death” state. The detailed Markov model is presented in Figure 1. At the same time, we made the following assumptions for the model: (1) Since the initial probability distribution of FD patients was not queried, we assumed that all patients entered the model in the state of “No symptoms”; (2) It was assumed that when a patient made a transfer to a state, he is only able to maintain this state or transfer to a state with a lower utility value, and could not return to a state with a higher utility value.

Based on the disease characteristics of FD and the administration of the relevant therapeutic regimen, we set the model cycle to 1 month, and the total timeframe of the model run was 3 years. Because changes in the state of a patient may occur at any time during a cycle, we corrected the model for half-cycling in order to more accurately simulate the process of state transfer in FD patients.

### 2.5 Costs, utility values, and transfer probability data

The cost data, utility values, and data sources used in this study are presented in Tables 1, 2, respectively. Since the healthcare system was chosen as the research perspective for this study, the costs included were direct medical costs. Direct healthcare cost is the cost of healthcare resources consumed by a treatment regimen, which in this study includes outpatient, pharmaceutical, and hospitalization costs. It was assumed that patients with “Multiple complications” required hospitalization. Data on transfer probabilities are based on previously published studies, and detailed data are available in the supplementary material. In terms of the transfer probability of “No symptoms” and “Acroparesthesia” to “Death”, we used the officially announced Chinese natural mortality rate of 7.9‰ in 2023. With regard to adverse events, we did not find specific data on utility reductions due to adverse events for either treatment regimen, and therefore did not consider additional costs and utility reductions due to them in this study.

**TABLE 1:** Cost inputs.

Cost	Cost value	Distribution	Source
Carbamazepine cost (¥/10 g)	40	gamma	315jiage.cn
Captopril cost (¥/2.5 g) Chlorosartan cost (¥/1.4 g) ALTA-a cost (¥/50 mg) Outpatient cost (¥/time) Hospitalization cost (¥/day)	14.3 14.8 4,880 12 376	gamma gamma gamma gamma gamma	yaofangwang.com www.menet.com www.hnysfww.com www.menet.com www.menet.com

**TABLE 2:** Utility inputs.

Markov state	Utility value	Distribution	Source
No symptoms	0.874	beta	Rombach et al. (2013)
Acroparesthesia	0.762	beta	Rombach et al. (2013)
Single complication	0.744	beta	Rombach et al. (2013)
Multiple complications	0.584	beta	Rombach et al. (2013)

### 2.6 Model outputs

The health outcome measure used in this study is quality-adjusted life years (QALYs). This measure is a standardized, universal health outcome measure that represents the number of years a patient would live in a fully healthy state. To reflect the cost difference between the two treatment options at the unit of effectiveness, we use the incremental cost-effectiveness ratio (ICER), which is the increase in cost for the “ERT” group relative to the “No ERT” group divided by the increase in QALYs. We used 1-3 times the World Health Organization’s recommended gross domestic product (GDP) *per capita* as the willingness-to-pay threshold. In 2023, the *per capita* GDP of China was ¥89,358. According to the Chinese Guidelines for Economic Evaluation of Pharmaceuticals (2020 Edition), the discount rate for both costs and benefits in this study was set at 5%.

### 2.7 Sensitivity analysis

We used one-way sensitivity analysis and probabilistic sensitivity analysis (PSA), respectively. The former is designed to detect the effect of a single parameter on ICER. The latter, on the other hand, simultaneously assesses the impact of uncertainty in all parameters on the results by performing 1000 random samples of each parameter in the model under different probability distributions, and then presenting the simulation results on the cost-effectiveness plane and plotting the cost-effectiveness acceptable curve. Both sensitivity analyses were able to test the stability of the test results.

### 2.8 Analysis software

All the above analytical procedures were carried out using TreeagePro2022 software (2022; TreeAge Software; Williamstown, Massachusetts).

### 2.9 Compliance with ethics guidelines

The data related to this study were obtained from clinical trials and previously published papers. This study did not contain any human or animal related experiments and therefore did not require approval from the Ethics Committee.

## 3 Results

### 3.1 Base-case results

The results of the base-case analysis results are shown in Table 3. In terms of health outputs, when QALY was used as a measure, the value for patients in the ERT group was 1.36, which was significantly higher than the QALY value for patients in the “No ERT” group (0.99). However, the ERT group also had to bear more costs. The ICER value derived from the deterministic analysis was ¥148,071.95/QALY, which is between one time the GDP *per capita* (¥89,358/QALY) and three times the GDP *per capita* (¥268,074/QALY). This indicates that ERT treatment is an economical program.

**TABLE 3:** Base-case results.

Treatment	Cost (¥)	Incr cost (¥)	Eff (QALY)	Incr eff (QALY)	ICER (¥/QALY)
No ERT	13078.97		0.99		
ERT	67532.16	54453.19	1.36	0.37	148071.95

### 3.2 One-way sensitivity analysis results

**FIGURE 2:**
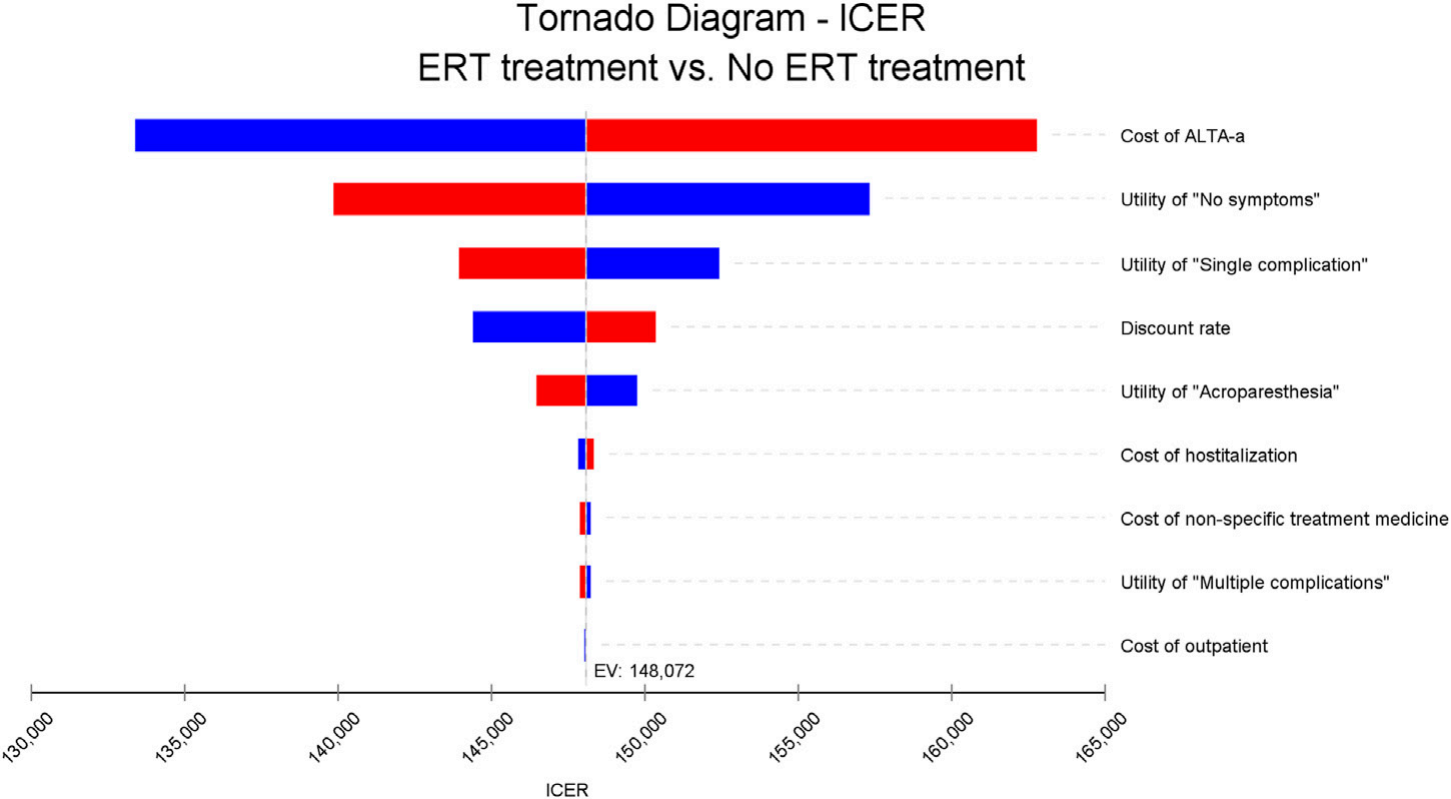
One-way sensitivity analysis results.

In order to test the stability of the results, this study conducted a one-way sensitivity analysis of the key parameters in the model. Specifically, it is presented by adjusting the key parameters upward as well as downward by 10% (the range of variation of the discount rate is 0%–8%), and finally the impact of the changes in the key parameters on the ICER is presented in the form of a tornado diagram (Figure 2). The different directional changes of the parameters in the figure are distinguished in red and blue color respectively. From the figure, it can be observed that the price of ALTA-a is the factor that has the greatest impact on the ICER, which reveals its important influence on the economics of the corresponding treatment program.

### 3.3 Probabilistic sensitivity analysis results

**FIGURE 3:**
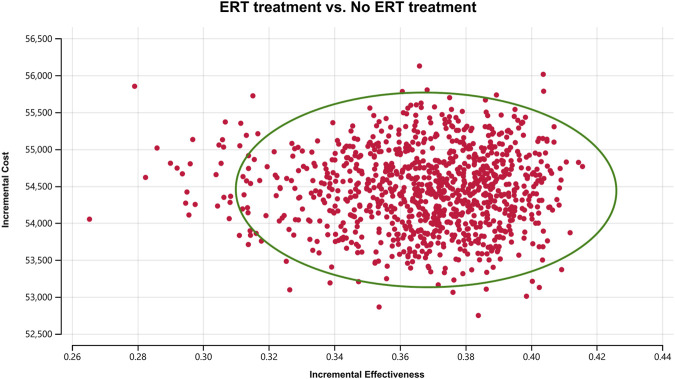
Cost-effectiveness plane for the ERT versus the “No ERT” group (WTP = ¥89358/QALY).

**FIGURE 4:**
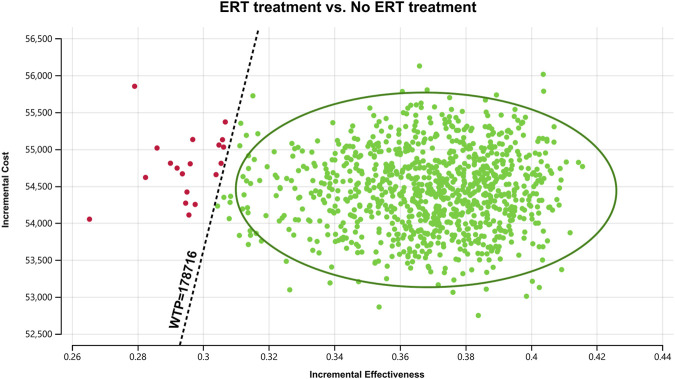
Cost-effectiveness plane for the ERT versus the “No ERT” group (WTP = ¥178716/QALY).

**FIGURE 5:**
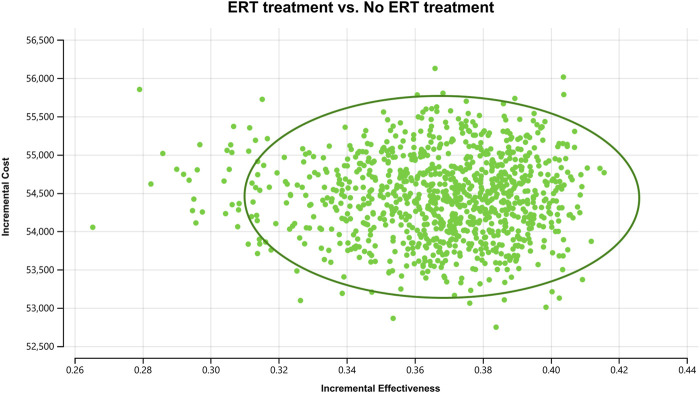
Cost-effectiveness plane for the ERT versus the “No ERT” group (WTP = ¥268074/QALY).

**FIGURE 6:**
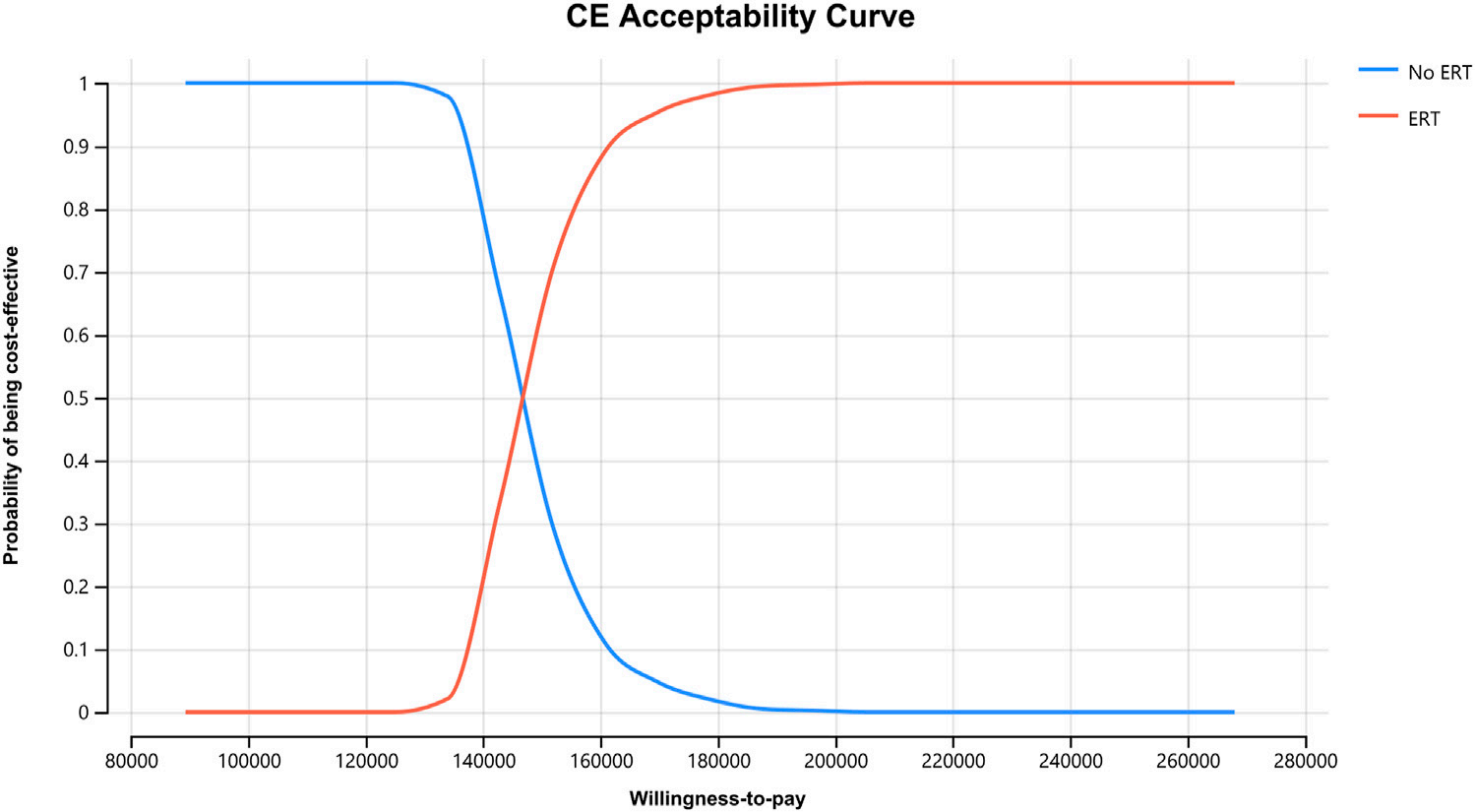
Cost-effectiveness acceptable curve.

In order to assess the impact of parameter uncertainty on the results, we performed a PSA. we used 1% and 10% of the mean as the estimated range of variation in standard deviation for the cost and utility values, respectively. The simulation results are presented on cost-effect quadrant plots as shown in Figures 3–5. The X-axis represents the incremental QALY obtained and the Y-axis represents the incremental cost. The green ellipse contains the 95% ICER estimate. We chose 1 and 3 times GDP *per capita* as thresholds for the analysis, respectively (to be able to visualize the trend of changes more closely, we also chose 2 times GDP *per capita* as a threshold). The results show that ERT treatment brings more costs as well as additional effects in all simulations. The average ICER value (¥148630.53/QALY) was similar to the results of the deterministic analysis (¥148071.95/QALY). In addition, we plotted the cost-effectiveness acceptable curve, and the results are shown in Figure 6. It shows the probability that two treatment programs have cost-effects at a given threshold. We can find that as the willingness-to-pay threshold is adjusted upward, the probability of having a cost-effect in the ERT group increases. The probability that the ERT group is cost-effective is 0% at a threshold of 1x GDP *per capita* (¥89,358/QALY), and this value rises to 98.1% and 100% when the threshold rises to 2x GDP *per capita* (¥178,716/QALY) and 3x GDP *per capita* (¥268,074/QALY), respectively.

## 4 Discussion

As a rare X chromosome-associated lysosomal storage disease, FD can lead to multiple organ diseases such as cardiac and renal diseases and even life-threatening complications, and its clinical manifestations are diversified and non-specific. FD is ranked 27th in China’s First Rare Disease Catalog, and even though the number of patients with FD is relatively small, the complexity of its clinical symptoms still needs to be taken seriously. The treatment of FD includes non-specific and specific treatment. Nonspecific treatment is the symptomatic management of each organ involvement. Specific therapy, on the other hand, regulates the activity of the corresponding enzymes and is targeted. Among them, ERT is a classical therapy. Several studies have confirmed the significant improvement of cardiac and renal functions of ALTA-a in FD patients (Anderson et al., 2014; Tang et al., 2021; Choi et al., 2008; Sasa et al., 2019; Lin et al., 2014; Ramaswami et al., 2019). Despite the excellent clinical performance, there are limited pharmacoeconomic studies on ERT for FD worldwide. Therefore, this study evaluated the economics of ERT for the treatment of FD patients in China from the perspective of the healthcare system.

In this study, we first performed a base-case analysis. The results showed that the ERT therapy group incurred higher costs and also obtained more QALYs. The ICER obtained by calculation was ¥148071.95/QALY, a value that is between 1 and 3 times the *per capita* GDP of China. This indicates that ERT therapy is an economical program. In a later unidirectional sensitivity analysis, we found that the cost of ALTA-a was the most important parameter leading to the change in ICER, which indicates that in the case of ERT therapy, the price of the drug is still the determining factor leading to the economics of the treatment program. Using PSA analysis, we found that the probability of the ERT treatment group being economical increases with the willingness-to-pay threshold, rising from 0% corresponding to 1x GDP *per capita* to 100% corresponding to 3x GDP *per capita*. We also assess the effect of parameter uncertainty on the results, demonstrating the stability of the results.

This study has several advantages. First, this study selected ERT, a classic and representative therapy, as a research subject, which will provide a reference for subsequent studies. Second, this study is the first pharmacoeconomic evaluation of ERT for the treatment of FD in China, which is useful for the selection of therapies for FD patients in China. Finally, this paper used a relatively novel Markov model, and the individual states can comprehensively reflect the disease progression of FD patients.

However, there are limitations to this study. First, due to the lack of data from the Chinese region and from FD-related clinical trials, some of the data used in the model of this study were derived from studies in other countries, which are likely not applicable to FD patients in China. In addition, due to the lack of reference standards, the effects of adverse reactions were not included in the model. However, the fact is that in clinical practice, prolonged injection of ALTA-a can lead to the formation of anti-drug antibodies (mostly IgG-type antibodies) that produce adverse reactions (van der Veen et al., 2019). These are likely to lead to an increase in the cost of treatment as well as a decrease in the associated utility values and have some impact on the final outcome. Secondly, due to the lack of relevant data, the distribution of costs as well as utilities in the PSA used mean values ± 1% and ±10%, respectively. The uncertainty of the real situation may be different from that reflected in the PSA of this study. Moreover, considering the ubiquity of ALTA-a at this stage, only ALTA-a therapy in ERT was considered in this study, however, ERT also includes other therapies such as ALTA-β. As a classical therapy for these diseases, a comprehensive pharmacoeconomic evaluation of ERT is necessary. Future studies can be conducted from other ERT therapies, which will strengthen the persuasive conclusions. Finally, this study still used the more traditional 1-3 times GDP *per capita* as the willingness-to-pay threshold, which is likely no longer applicable to diseases such as FD. We believe that the setting of willingness-to-pay thresholds for drugs related to rare diseases is an important aspect of future pharmacoeconomic evaluations. We recognize that the above limitations may lead to very different conclusions. However, based on the limited data available, we believe that all data in this study were obtained from the best available sources, minimizing the impact of data bias on obtaining realistic conclusions.

Our study applied Markov modeling, which has distinct advantages and disadvantages. It has been pointed out that in the field of pharmacoeconomic analysis, Markov modeling is particularly suitable for diseases involving duration risk, including the risk of death (Carta and Conversano, 2020), which fits well with the disease characteristics of FD. In terms of application, it can predict future state shifts, which helps relevant decision making. However, its disadvantages cannot be ignored. The first is that additions to the modeled states will become complex. If the FD disease states are further refined and more disease states are obtained in the future, the transfer matrix will be large and difficult to manage. At the same time, the model is highly dependent on assumptions and ignores the interference of other external factors, which can affect the accuracy of the study. As FD studies continue to be conducted, we believe that dynamic modeling on pharmacoeconomic evaluation is likely to be applied in future FD-related studies. As new clinical evidence continues to emerge, such dynamic modeling will provide a clearer and more specific perspective on the innovation of FD therapies.

In addition, based on real-world data, ERT is effective in slowing down disease progression (Beck et al., 2022; Parini et al., 2020). At the same time, it is associated with high costs and burden of healthcare resource utilization (Katsigianni and Petrou, 2022; Jovanovic et al., 2024). This corresponds to the results of our study. Analyzing the real-world data in conjunction with the expected results of our model can provide a more objective perspective.

It is worth noting that despite the fact that ALTA-a has been included in the health insurance catalog and the cost of medication has dropped significantly, there will still be some patients who will not be able to afford this portion of the expenditure. This will have a greater impact when patients consider ERT treatment. We agree with the recommendation made by previous scholars, that is, we suggest the relevant drug manufacturers to implement “patient assistance programs” to improve the accessibility of the drug (Xiang et al., 2023), so that more patients can obtain more effective treatment.

In addition, due to the diversity of clinical symptoms of FD, patients with FD are often delayed in diagnosis, resulting in failure to receive timely and effective treatment (Mehta et al., 2010). Therefore, starting ERT as early as possible or at the right time may have a positive impact on the economics of the relevant ERT treatment regimen. This also requires a concerted effort between patients and clinicians in terms of disease awareness. In addition, because China’s economic development is highly unbalanced, there are large differences in the level of economic development between regions. Therefore, the results of this study should be considered in the context of local economic development in different regions. In economically developed regions, such as Beijing and Shanghai (with GDP *per capita* of $27,729 and $26,252, respectively, which are both more than twice the national GDP *per capita* (Xiang et al., 2023)), the probability of ERT having a cost-effectiveness will be higher. Conversely, this probability decreases in regions with relatively poor economic development. Finally, we call for the emergence of more clinical data based on FD patients in Chinese regions, which will provide more reliable evidence for the economic evaluation of FD treatment and facilitate more accurate study results. Although the cost-effectiveness of ERT treatment was demonstrated in our study, the patients’ quality of life and the economic burden still need to be emphasized (Jovanovic et al., 2024). For example, as suggested by some scholars, home infusion and self-administration may help to reduce the burden associated with ERT (Milligan et al., 2006; Beck et al., 2013).

It has already been mentioned that the life expectancy of FD patients, especially men, is significantly lower. In context (timeliness of treatment and misdiagnosis, etc.), the actual life expectancy of these patients may be even lower. In the future, the clinical diagnosis of FD patients will be crucial, as the clinical phenotype of FD is extensive and heterogeneous, as the same genotype is not identical in the same family, and the detection of mutation sites of unknown significance is unsupported by, or does not correspond to clinical data, which creates multiple complexities in clinical diagnosis and treatment. Therefore, the development of individualized diagnosis and treatment plans for FD is very important in the future, and requires the relevant healthcare professionals to have a full understanding of the disease characteristics, progression patterns, and socio-economic conditions of patients with FD. In addition, besides ERT, molecular chaperone therapy is also a popular therapy for FD. Among them, the molecular chaperone with more clinical use is migalastat, which is suitable for patients with *GLA* missense mutations that retain some α-Gal A activity. Currently, it is not yet available in China. Relevant studies incorporating real-world data from the Chinese healthcare system are one aspect that could be pursued in future FD-related studies, especially if there is sufficient local Chinese data for these popular therapies. The availability of more evidence of economic evaluation based on the Chinese context will strengthen the conclusions we have drawn and will help to customize future pharmacoeconomic models.

## 5 Conclusion

In summary, ERT treatment is the economically viable option from the perspective of the healthcare system. This study will help relevant organizations in their decision-making process, and the excellent efficacy, safety, and affordability of ERT are likely to make it a dominant treatment for FD in the future.

## Data Availability

The original contributions presented in the study are included in the article/Supplementary Material, further inquiries can be directed to the corresponding author.
