# Supplementary material for: Cost-effectiveness analysis of enzyme replacement therapy for the treatment of Chinese patients with fabry disease: a Markov model
Source: Front Pharmacol. 2025 Mar 11;16:1546018. doi: 10.3389/fphar.2025.1546018 (PMC11932859; doi:10.3389/fphar.2025.1546018)
Supplement: Supplementary file 1 [file DataSheet1.docx]

| Transition | Probability |
| --- | --- |
| “No symptoms” to “No symptoms” | 0.7792 |
| “No symptoms” to “Acroparesthesia” | 0.1442 |
| “No symptoms” to “Single symptom” | 0.0687 |
| “No symptoms” to “Death” | 0.0079 |
| “Acroparesthesia” to “Acroparesthesia” | 0.8174 |
| “Acroparesthesia” to “Single complication” | 0.1747 |
| “Acroparesthesia” to “Death” | 0.0079 |
| “Single complication” to “Single complication” | 0.8797 |
| “Single complication” to “Multiple symptoms” | 0.0642 |
| “Single complication” to “Death” | 0.0561 |
| “Multiple symptoms” to “Multiple symptoms” | 0.5932 |
| “Multiple symptoms” to “Death” | 0.4068 |

Table 1. Transition probabilities of “No ERT” group.

| Transition | Probability |
| --- | --- |
| “No symptoms” to “No symptoms” | 0.8700 |
| “No symptoms” to “Acroparesthesia” | 0.0830 |
| “No symptoms” to “Single symptom” | 0.0391 |
| “No symptoms” to “Death” | 0.0079 |
| “Acroparesthesia” to “Acroparesthesia” | 0.8563 |
| “Acroparesthesia” to “Single symptom” | 0.1358 |
| “Acroparesthesia” to “Death” | 0.0079 |
| “Single symptom” to “Single symptom” | 0.9222 |
| “Single symptom” to “Multiple symptoms” | 0.0415 |
| “Single symptom” to “Death” | 0.0363 |
| “Multiple symptoms” to “Multiple symptoms” | 0.7135 |
| “Multiple symptoms” to “Death” | 0.4068 |

Table 2. Transition probabilities of “ERT” group.
